# Episodic Memory in Older Adults Without Dementia: A Meta-Analysis of Aerobic Exercise Interventions

**DOI:** 10.1093/geroni/igab046.1768

**Published:** 2021-12-17

**Authors:** Sarah Aghjayan, Themistokles Bournias, Chelsea Stillman, Shannon Donofry, Anna Marsland, Kirk Erickson

**Affiliations:** 1 University of Pittsburgh, Pittsburgh, Pennsylvania, United States; 2 Carnegie Mellon University, Carnegie Mellon University, Pennsylvania, United States; 3 University of Pittsburgh, University of Pittsburgh, Pennsylvania, United States

## Abstract

The effect of aerobic exercise interventions on episodic memory performance among older adults without dementia remains a matter of intense debate. Prior meta-analyses examining this association have reported minimal improvements in episodic memory performance following exercise training but have also been plagued by several limitations, including restrictive inclusion criteria, combined sample populations, and infrequent examination of the effect of exercise parameters (e.g., volume). To address these gaps, we conducted a meta-analysis of randomized controlled trials (RCTs) to determine if aerobic exercise interventions influence episodic memory performance in older adults without dementia and to examine potential moderators of these effects (e.g., sample and intervention characteristics). Included studies met the following criteria: (1)Studies of adults (M≥55 years) with normal cognition, subjective cognitive decline, or mild cognitive impairment; (2)Aerobic exercise RCTs; and (3)Assessment of episodic memory. Intervention effects were represented by Hedges’ g and combined into pooled effect sizes using random- and mixed-effects models. Thirty-three studies met inclusion criteria, representing data from 2,488 participants. The primary analysis yielded a significant positive effect of aerobic exercise on episodic memory (Hedges’ g[CI]=0.28[0.10-0.47]; p=0.003). Mixed-effects analyses demonstrated a positive effect on episodic memory among studies with a high percentage of females (>66%), participants with normal cognition, studies reporting intensity, studies with a no-contact or nonaerobic physical activity control group, and studies prescribing 2,100–3,900 total minutes of activity (range 540–8,190 minutes). These results suggest that aerobic exercise may act as an accessible, non-pharmaceutical intervention to improve episodic memory in late adulthood before changes in cognition are detected.

